# Comparison of Two 16S rRNA Primers (V3–V4 and V4–V5) for Studies of Arctic Microbial Communities

**DOI:** 10.3389/fmicb.2021.637526

**Published:** 2021-02-16

**Authors:** Eduard Fadeev, Magda G. Cardozo-Mino, Josephine Z. Rapp, Christina Bienhold, Ian Salter, Verena Salman-Carvalho, Massimiliano Molari, Halina E. Tegetmeyer, Pier Luigi Buttigieg, Antje Boetius

**Affiliations:** ^1^Alfred Wegener Institute for Polar and Marine Research, Bremerhaven, Germany; ^2^Max Planck Institute for Marine Microbiology, Bremen, Germany; ^3^School of Oceanography, University of Washington, Seattle, WA, United States; ^4^Faroe Marine Research Institute, Tórshavn, Faroe Islands; ^5^Department of Microbiology, Morrill Science Center IVN, University of Massachusetts, Amherst, MA, United States; ^6^Center for Biotechnology, Bielefeld University, Bielefeld, Germany; ^7^MARUM, University of Bremen, Bremen, Germany

**Keywords:** microbial communities, amplicon sequencing, method comparison, universal primers, Arctic Ocean, molecular observatory

## Abstract

Microbial communities of the Arctic Ocean are poorly characterized in comparison to other aquatic environments as to their horizontal, vertical, and temporal turnover. Yet, recent studies showed that the Arctic marine ecosystem harbors unique microbial community members that are adapted to harsh environmental conditions, such as near-freezing temperatures and extreme seasonality. The gene for the small ribosomal subunit (16S rRNA) is commonly used to study the taxonomic composition of microbial communities in their natural environment. Several primer sets for this marker gene have been extensively tested across various sample sets, but these typically originated from low-latitude environments. An explicit evaluation of primer-set performances in representing the microbial communities of the Arctic Ocean is currently lacking. To select a suitable primer set for studying microbiomes of various Arctic marine habitats (sea ice, surface water, marine snow, deep ocean basin, and deep-sea sediment), we have conducted a performance comparison between two widely used primer sets, targeting different hypervariable regions of the 16S rRNA gene (V3–V4 and V4–V5). We observed that both primer sets were highly similar in representing the total microbial community composition down to genus rank, which was also confirmed independently by subgroup-specific catalyzed reporter deposition-fluorescence *in situ* hybridization (CARD-FISH) counts. Each primer set revealed higher internal diversity within certain bacterial taxonomic groups (e.g., the class *Bacteroidia* by V3–V4, and the phylum *Planctomycetes* by V4–V5). However, the V4–V5 primer set provides concurrent coverage of the archaeal domain, a relevant component comprising 10–20% of the community in Arctic deep waters and the sediment. Although both primer sets perform similarly, we suggest the use of the V4–V5 primer set for the integration of both bacterial and archaeal community dynamics in the Arctic marine environment.

## Introduction

The Arctic Ocean is the most rapidly changing marine region on the planet due to its fast warming causing substantial sea-ice loss (Peng and Meier, 2018; Dai et al., 2019), as well as increasing pollution (Peeken et al., 2018). To assess the impact of global climate change on marine food web dynamics and elemental cycles, it is important to monitor variations in microbial community structure with time (Karl and Church, 2014; Fuhrman et al., 2015; Buttigieg et al., 2018). However, the Arctic Ocean is generally under-sampled in ice-covered regions and in winter (Wassmann et al., 2011), particularly with regard to assessments of its microbial communities and their biogeochemical functions (Boetius et al., 2015). Until recently, microbial monitoring efforts in the deep Arctic Ocean consisted of 1 year-round long-term time series at the HAUSGARTEN observatory in the Fram Strait (Soltwedel et al., 2005, 2015), as well as a few other process studies (e.g., Kirchman et al., 2007; Alonso-Sáez et al., 2008; Nikrad et al., 2012; Wilson et al., 2017; Müller et al., 2018).

The Arctic Ocean features substantial vertical structure that may select for specific microbial types in the sea ice (Boetius et al., 2015; Rapp et al., 2018), in the ice-free and the ice-covered highly stratified surface waters (Wilson et al., 2017; Fadeev et al., 2018), in the sinking particles (further addressed as “marine snow”; Fadeev et al., 2020), as well as in the water and sediments of the deep-sea where temperatures are year-round close to freezing point temperatures (Bienhold et al., 2012; Hoffmann et al., 2017; Wilson et al., 2017; Rapp et al., 2018; Fadeev et al., 2020). Throughout the annual cycle, Arctic surface waters bacterial and archaeal communities exhibit pronounced fluctuations of the dominant taxonomic groups (Alonso-Sáez et al., 2008; Wilson et al., 2017; Müller et al., 2018), which are strongly associated with presence of sea ice and the seasonal phytoplankton blooms (Kirchman et al., 2007; Nikrad et al., 2012; Fadeev et al., 2018; Cardozo-Mino et al., 2020). In winter, as well as under ice-covered conditions, the communities are dominated by the bacterial classes *Alphaproteobacteria* (mainly the SAR11 clade), *Dehalococcoidia* (mainly SAR202 clade), and the archaeal class *Nitrososphaeria* (Alonso-Sáez et al., 2008; Wilson et al., 2017; Müller et al., 2018). In the summer, and under ice-free conditions, the communities are dominated by the bacterial classes *Bacteroidia* (mainly the order *Flavobacteriales*) and *Gammaproteobacteria* (mainly the orders *Alteromonadales* and *Oceanospirillales*; Wilson et al., 2017; Fadeev et al., 2018). During the summer, differences between ice-covered and ice-free communities also affect the microbial diversity of the deep ocean and the seafloor *via* alterations of microbial communities on marine snow (Fadeev et al., 2020).

In the framework of the FRAM Microbial Observatory (FRontiers in Arctic marine Monitoring), we are aiming to develop a standardized methodology for long-term observations of microbial communities in these highly diverse Arctic Ocean environments, which will be also comparable to other long-term microbial time series locations (e.g., HOT and BATS). Unlike other time series sites of the world, the ice-cover and the harsh conditions of the Arctic Ocean are limiting the accessibility of the sampling sites to the summer months. Sampling campaigns during the winter (when microbial biomass is low; Kirchman et al., 2007; Alonso-Sáez et al., 2008) are rare and have only recently been achieved using autonomous samplers with limited sampling capacities (Liu et al., 2020). Therefore, the unique conditions and the currently available technologies constrain year-round microbial observations to PCR-based approaches (i.e., 16S rRNA gene amplicon sequencing), which can be realized with low concentrations of DNA (Thomas et al., 2012). Metagenomics approaches suggest that the functional capacity of marine microbial communities is strongly linked to their taxonomic composition (Galand et al., 2018; McNichol et al., 2020). Thus, when supported by curated taxonomic databases (e.g., SILVA 16S rRNA gene reference; Quast et al., 2013), 16S rRNA gene amplicon sequencing provides an affordable high-throughput tool for addressing traditional community ecology questions, especially under the constrained sampling conditions of the Arctic marine environment.

A critical step in 16S rRNA gene sequencing studies is the selection of PCR primers for DNA amplification (Armougom, 2009; Wang and Qian, 2009). Throughout the years, many primer sets were designed for diversity studies of specific taxonomic groups (e.g., SAR11 clade; Apprill et al., 2015), and attempts have been made to develop a more universal 16S rRNA gene primer sets that could cover close to the entire diversity of a natural microbial community (e.g., Earth Microbiome Project; Caporaso et al., 2012; Gilbert et al., 2014). The development of primer sets for the amplification of 16S rRNA genes is conducted *in silico* using reference databases (e.g., Klindworth et al., 2013). The Arctic Ocean is the smallest and shallowest of all five oceans, representing 4% of the area and 1% of the volume of the global ocean. Nevertheless, it plays an important role in global processes that are strongly affected by the ongoing climatic changes and is considered relevant for several Earth System tipping points (Wassmann and Reigstad, 2011; Lenton et al., 2019). Furthermore, being the coldest among the oceans, with strong stratification and only limited deep-water exchange, the Arctic Ocean is likely to contain unique endemic microbial diversity that drives its biogeochemical cycles (Kirchman et al., 2009; Ghiglione et al., 2012; Pedrós-Alió et al., 2015). An example for such locally adapted Arctic diversity was recently found with Arctic specific members of the ubiquitous SAR11 clade (Kraemer et al., 2020). Furthermore, despite its global importance, sampling effort in the Arctic Ocean is low, especially in and under the sea ice and in the deep basin, as well as generally during the wintertime (Wassmann et al., 2011; Royo-Llonch et al., 2020). Thus, the reference databases are likely lacking proper coverage of the complexity and dynamics of the Arctic Ocean microbiomes that may result in biased representations of them by currently available 16S rRNA gene primers.

One of the most extensively used primer set for the investigation of bacterial diversity in various environments is the 341F/785R (targeting the V3–V4 hypervariable regions of the 16S rRNA gene) that was developed by Klindworth et al. (2013). For the investigation of marine microbiomes, an alternative primer set 515F-Y/926R (targeting the V4–V5 hypervariable regions of the 16S rRNA gene), which is also able to capture the diversity of the archaeal communities, has been developed by Parada et al. (2016). Currently, both V3–V4 and V4–V5 primer sets are widely used in studies of marine microbial communities and were extensively tested using mock and natural communities of temperate waters (e.g., Wear et al., 2018; Willis et al., 2019; McNichol et al., 2020). However, no study has systematically tested the performance of these primer sets on microbial communities of the Arctic Ocean.

In an attempt to select the most suitable primer set for the long-term monitoring of Arctic microbial communities as part of the FRAM Molecular Observatory, we present here a performance comparison of the 16S rRNA gene primer sets V3–V4 (341F/785R) and V4–V5 (515F-Y/926R). Our hypothesis was that due to relatively low representation of Arctic microbial communities in public databases (due to low number of existing studies), the 16S rRNA gene primer sets may capture different parts of microbial diversity in these unique environments. To test this hypothesis, we have conducted a direct comparison of the taxonomic coverage and potential biases of the two primer sets in 37 field samples collected from various environments of the Arctic Ocean, including sea-ice, surface and deep water column, marine snow, and deep-sea sediment. As an independent line of validation, we performed cell counting of five key taxonomic subgroups in a subset of the field samples *via* CARD-FISH (catalyzed reporter deposition-fluorescence *in situ* hybridization).

## Materials and Methods

### Sample Collection

The samples included in this study were collected at the long-term ecological research (LTER) site HAUSGARTEN in Fram Strait and the central Arctic Ocean (Supplementary Figure 1 and Supplementary Table 1). The samples were collected as follows:

• The sea-ice cores were collected using an ice corer (9 cm diameter; Kovacs Enterprise, Roseburg, OR, United States) and broken into subsections to facilitate quicker melting. The lower 30–50 cm of the sea ice (depending on total core length) was melted in plastic containers (rinsed with ethanol and ultrapure water) at 4°C in the dark. The melting of the sea ice took ∼24 h and the samples were immediately filtered on 0.22 μm Sterivex^TM^ membranes as soon as the last piece of sea ice melted. Additional samples for microscopy counts were filtered onto 0.22 μm polycarbonate membranes (Whatman Nucleopore, Buckinghamshire, United Kingdom), with sterile filtered formalin at a final concentration of 2% and stored at −20°C.
• The water sampling was carried out using 12 L Niskin bottles mounted on a CTD rosette (Sea-Bird Electronics Inc., SBE 911 plus probe, Bellevue, WA, United States) and filtered on 0.22 μm Sterivex^TM^ membranes. The Sterivex^TM^ membranes were then stored at −20°C until further processing. Additional samples for microscopy counts were filtered onto 0.22 μm polycarbonate membranes (Whatman Nucleopore, Buckinghamshire, United Kingdom), with sterile filtered formalin at a final concentration of 2% and stored at −20°C.
• The deep-sea sediment cores were retrieved by a TV-guided multicorer, and subsamples of the uppermost centimeter of the cores were collected with syringes and immediately stored at −20°C until further processing.
• The marine snow samples were collected using sediment traps of the long-term moorings at the LTER site HAUSGARTEN (Bauerfeind et al., 2009; Lalande et al., 2013). Collection cups (400 ml) were filled with filtered seawater, adjusted to a salinity of 40 and poisoned with HgCl_2_ (0.14% final solution) to preserve samples during deployment and after recovery (Metfies et al., 2017). After recovery, samples were stored at +4°C, swimmers were removed and samples were split by a wet splitting procedure (Bodungen et al., 2013). In this study, we used 1/32 splits of the original trap sample. Sinking particles from the sediment trap samples were collected on 0.22 μm Sterivex filters and stored at −20°C.

All metadata of the samples are accessible *via* the Data Publisher for Earth and Environmental Science PANGAEA^1^, the PANGAEA event IDs are listed in Supplementary Table 1. Sampling map was produced using Ocean Data View v5.2.1 (Schlitzer, 2018).

### DNA Isolation and 16S rRNA Gene Amplicon Sequencing

Genomic DNA was isolated in a combined chemical and mechanical procedure using the PowerWater DNA Isolation Kit for sea ice, water, and sediment traps and using the PowerSoil DNA Isolation Kit for sediment samples (MO BIO Laboratories, Inc., Carlsbad, CA, United States). Prior to DNA isolation, the 0.22 μm Sterivex^TM^ membrane cartridges of the seawater and sea ice samples were cracked open in order to place the filters into the kit-supplied bead beating tubes. The isolation was continued according to the manufacturer’s instructions, and DNA was stored at −20°C. Library preparation was performed according to the standard instructions of the 16S Metagenomic Sequencing Library Preparation protocol (Illumina^TM^, Inc., San Diego, CA, United States). Two different hypervariable regions of the bacterial 16S rRNA gene were amplified using aliquots of the isolated DNA from each sample. The V3–V4 region was amplified using the S-D-Bact-0341-b-S-17 (5′-CCTACGGGNGGCWGCAG-3′) and the S-D-Bact-0785-a-A-21 (5′-GACTACHVGGGTATCTAATCC-3′) primers (Klindworth et al., 2013). The V4–V5 regions was amplified using the 515F-Y (5′-GTGYCAGCMGCCGCGGTAA-3′) and the 926R (5′-CCGYCAATTYMTTTRAGTTT-3′) primers (Parada et al., 2016). Sequences were obtained on the Illumina MiSeq^TM^ platform in a 2 × 300 bp paired-end run and for surface water samples on the Illumina HiSeq^TM^ platform in a 2 × 250 bp paired-end run (CeBiTec, Bielefeld, Germany), following the standard instructions of the 16S Metagenomic Sequencing Library Preparation protocol.

Raw paired-end, primer-trimmed reads were deposited in the European Nucleotide Archive (ENA; Harrison et al., 2019) under accession number PRJEB31938. The data were archived using the brokerage service of the German Federation for Biological Data (GFBio; Diepenbroek et al., 2014).

### Bioinformatics and Statistical Analyses

The raw paired-end reads were primer-trimmed using cutadapt (Martin, 2011). Further analyses were conducted using R v4.0.0^2^ in RStudio v1.2.5042^3^. The libraries were processed using DADA2 v1.16 (Callahan et al., 2016a), following the suggested workflow (Callahan et al., 2016b). The reads in MiSeq libraries were truncated at 255 bp length for forward reads and at 200 bp length for reverse reads, to facilitate the technical quality drop at the end of the reads. Reads in both MiSeq and HiSeq were then trimmed for low-quality bases and merged based on a minimum overlap of 10 bp. Chimeras and amplicon sequence variants (ASVs) that were observed in only one sample were filtered out. The representative sequences were taxonomically classified against SILVA 16S rRNA gene reference database release 138 (Quast et al., 2013; Yilmaz et al., 2014). The ASVs that were taxonomically unclassified at phylum rank or were not assigned to bacterial or archaeal lineages were excluded from further analysis. Furthermore, all ASVs that were taxonomically assigned to mitochondria and chloroplast were removed from the dataset.

Sample data matrices were managed using the R package “phyloseq” v1.32 (McMurdie and Holmes, 2013), and plots were generated using the R package “ggplot2” v3.3.0 (Gómez-Rubio, 2017). The sample rarefaction analyses were conducted using the R package “iNEXT” v2.0.20 (Hsieh et al., 2016). To test the effect of the different primer sets on the taxonomic composition of the microbial communities, as well as to test for differences between microbial communities of different types of samples, a two-way permutation multivariate analysis of variance (“Two-way PERMANOVA”) of Jensen–Shannon Divergence distance matrix was conducted (using the function “adonis2” in the R package “vegan” v.2.5.6; Oksanen et al., 2007).

Scripts for processing data can be accessed at https://github.com/edfadeev/Arctic-16S-Primers-comparison/.

### Catalyzed Reporter Deposition Fluorescence *in situ* Hybridization

Both sea ice and seawater samples were directly fixed in 4% formalin for 4 h at 4°C, filtered onto 0.22 μm polycarbonate Track-Etched^TM^ membranes (Whatman Nucleopore, Buckinghamshire, United Kingdom), and stored at −20°C. The CARD-FISH was applied based on the protocol established by Pernthaler et al. (2002), using horseradish-peroxidase (HRP)–labeled oligonucleotide probes (^4^ Ulm, Germany; Supplementary Table 4). All probes were checked for specificity and coverage of their target groups against the SILVA 16S rRNA gene reference. All filters were embedded in 0.2% low-gelling-point agarose and treated with 10 mg mL^–1^ lysozyme solution (Sigma-Aldrich Chemie GmbH, Hamburg, Germany) for 1 h at 37°C. Subsequently, endogenous peroxidases were inactivated by submerging the filter pieces in 0.15% H_2_O_2_ in methanol for 30 min, before rinsing in Milli-Q water and dehydration in 96% ethanol. Then, the filters were covered in a hybridization buffer and a probe concentration of 0.2 ng μL^–1^. Hybridization was performed at 46°C for 2.5 h, followed by washing in a pre-warmed washing buffer at 48°C for 10 min, and 15 min in 1x PBS. Signal amplification was carried out for 45 min at 46°C with an amplification buffer containing either tyramide-bound Alexa 488 (1 μg/mL^–1^) or Alexa 594 (0.33 μg mL^–1^). Afterward, the cells were counterstained in 1 μg/mL^–1^ DAPI (4′,6-diamidino-2-phenylindole; Thermo Fisher Scientific GmbH, Bremen, Germany) for 10 min at 46°C. After rinsing with Milli-Q water and 96% ethanol, the filter pieces were embedded in a 4:1 mix of Citifluor (Citifluor Ltd., London, United Kingdom) and Vectashield (Vector Laboratories, Inc., Burlingame, United States) and stored overnight at −20°C for later microscopy evaluation.

### Automated Image Acquisition and Cell Counting

The filters were evaluated microscopically under a Zeiss Axio Imager.Z2 stand (Carl Zeiss MicroImaging GmbH, Jena, Germany), equipped with a multipurpose fully automated microscope imaging system (MPISYS), a Colibri LED light source illumination system, and a multi-filter set 62HE (Carl Zeiss MicroImaging GmbH, Jena, Germany). Pictures were taken *via* a cooled charged-coupled-device (CCD) camera (AxioCam MRm; Carl Zeiss AG, Oberkochen, Germany) with a 63x oil objective, a numerical aperture of 1.4, and a pixel size of 0.1016 μm/pixel, coupled to the AxioVision SE64 Rel.4.9.1 software (Carl Zeiss AG, Oberkochen, Germany) as described by Bennke et al. (2016). Exposure times were adjusted after manual inspection with the AxioVision Rel.4.8 software coupled to the SamLoc 1.7 software (Zeder et al., 2011), which was also used to define the coordinates of the filters on the slides. For image acquisition, channels were defined with the MPISYS software, and a minimum of 55 fields of view with a minimum distance of 0.25 mm were acquired of each filter piece by recording a *z*-stack of seven images in autofocus.

Cell enumeration was performed with the software Automated Cell Measuring and Enumeration Tool (ACMETool3, 2018-11-09; M. Zeder, Technobiology GmbH, Buchrain, Switzerland). Cells were counted as objects according to manually defined parameters separately for the DAPI and FISH channels.

## Results and Discussion

**FIGURE 1:**
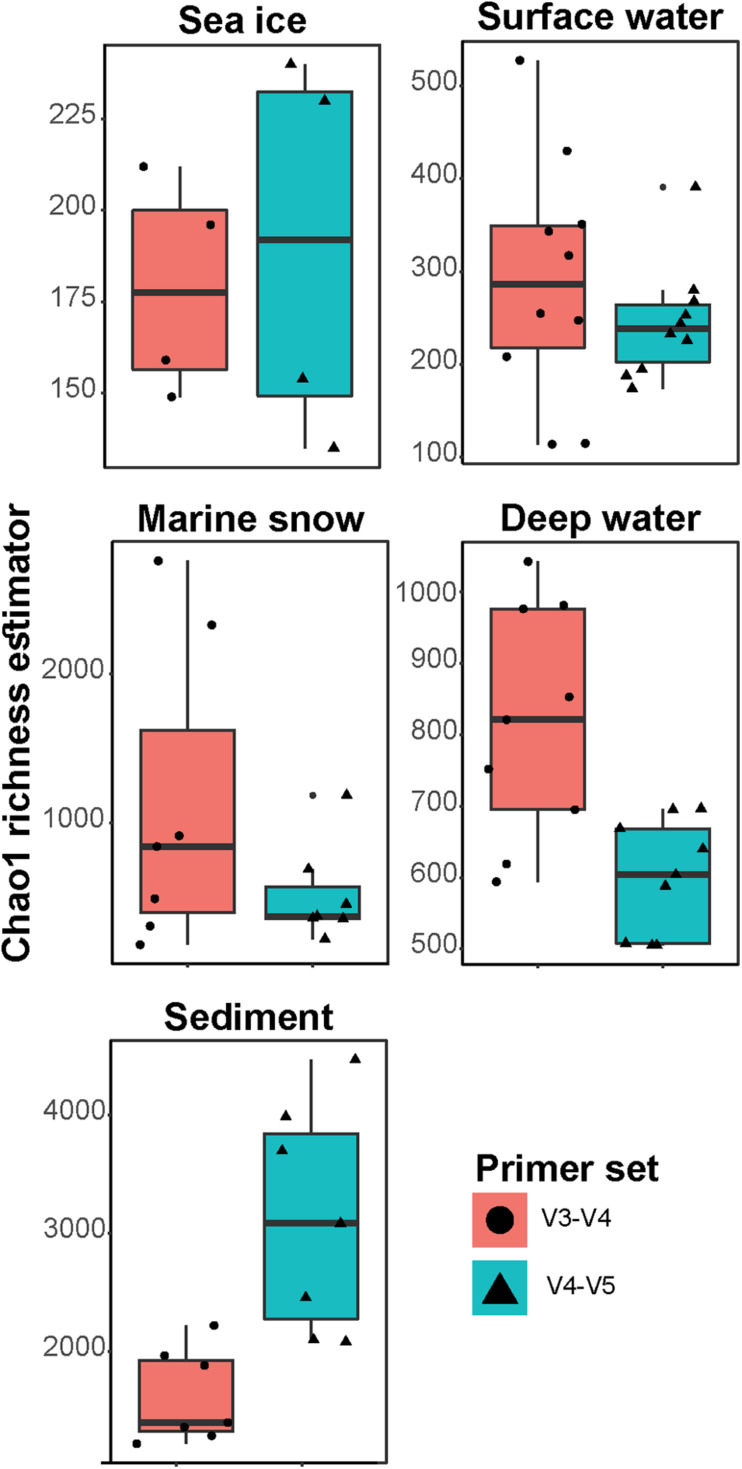
Chao1 richness estimates in the different sample types. Different primer sets represented by colors and shapes. Please note the differences of *y*-axis between the panels.

In this study, aliquots of 37 DNA samples from different environments in the Arctic Ocean (sea ice, surface and deep ocean water, marine snow, and seafloor sediment; Supplementary Table 1) were sequenced using two common primers sets that amplify either the V3–V4 or the V4–V5 hypervariable regions in the 16S rRNA gene and were subjected to the same bioinformatic workflow. Both primer sets showed a similar decrease in the number of sequences throughout the workflow, with 62 ± 13% and 68 ± 9% of sequences retained per sample, respectively. The final datasets consisted of 3,318,649 sequences in the V3–V4 dataset that were assigned to 12,045 ASVs and 3,340,628 sequences in the V4–V5 dataset that were assigned to 14,505 ASVs (Supplementary Table 2). In addition, the ASVs which were taxonomically assigned to eukaryotic, mitochondrial or chloroplast sequences, as well as ASVs unclassified at phylum rank, were also removed from further analysis (ca. 9% and ca. 17% of sequences in V3–V4 and V4–V5 datasets, respectively). In both datasets, an asymptotic extrapolation of the rarefaction curves did not further increase the number of observed ASVs (Supplementary Figure 2). Although, most likely further microbial diversity remains to be uncovered in all sampled environments, the rarefaction curves suggest that our samples contained most of the potential community richness covered by both primer sets. In sea ice, surface water (<30 m depth) and marine snow, both primer sets showed similar community richness (Figure 1). However, in the deep-water communities (>600 m depth), richness was significantly different between the primer sets (Wilcoxon Signed-Rank Test; *p* < 0.01), with ca. 40% more bacterial ASVs in the V3–V4. In contrast, the sediment community richness was significantly higher in the V4–V5 dataset (Wilcoxon signed-rank test; *p* < 0.01), with up to double the amount of bacterial ASVs compared to the V3–V4 dataset. The main taxonomic groups, typically observed in the Arctic marine environment, such as the classes *Alphaproteobacteria*, *Bacteroidia*, and *Gammaproteobacteria*, dominated both datasets (each comprising 10–30% of sequences in V3–V4 and V4–V4 datasets, respectively). However, within these groups significant differences between datasets in the number of observed ASVs were detected.

**FIGURE 2:**
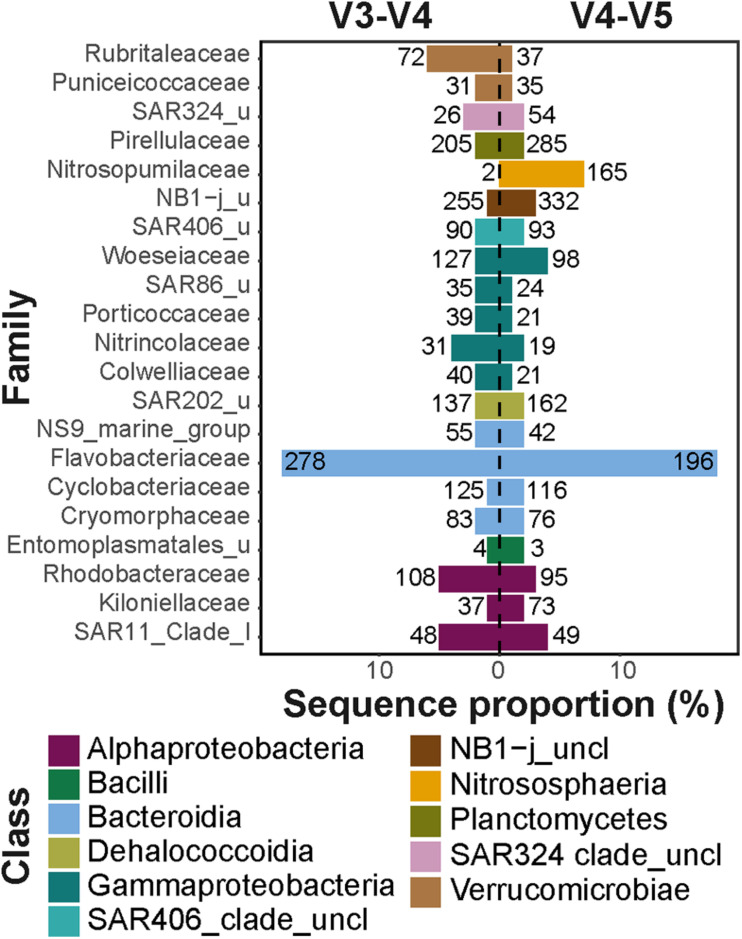
Major taxonomic families in V3–V4 and V4–V5 datasets. The *x*-axis represents the total sequence proportion of each family in V3–V4 (left panel) and V4–V5 (right panel) datasets. The numbers at each column represent the number of observed ASVs affiliated with each taxonomic family. Different taxonomic classes are represented by color code. Only families that comprised at least 1% of sequences in at least one of the datasets were included in the visualization.

In the V3–V4 dataset, the *Bacteroidia* and *Gammaproteobacteria* showed the highest differences in number of observed ASVs within each class (i.e., type richness) compared to the V4–V5 dataset (Supplementary Table 2). The family *Flavobacteriaceae* (class *Bacteroidia*) comprised 18% of all sequences in both datasets; however, in the V3–V4 dataset, it consisted of one third more ASVs compared to the V4–V5 dataset (total of 278 and 196 ASVs, respectively; Figure 2). This difference in the number of observed ASVs was mainly associated with ASVs of the genus *Polaribacter* (total of 28 and 14 ASVs, respectively), a key heterotrophic bacterium that responds to phytoplankton blooms in mid- and high-latitudes (Gómez-Pereira et al., 2010; Fadeev et al., 2018; Avcı et al., 2020). The orders *Alteromonadales*, *Cellvibrionales*, and *Oceanospirillales* (all within the class *Gammaproteobacteria*), which comprised 4–6% of all sequences in the V3–V4 dataset and 3% of all sequences in the V4–V5 dataset, also showed differences between datasets in the number of observed ASVs (Supplementary Table 3). Each of these *Gammaproteobacteria* orders contained two times more ASVs in the V3–V4 dataset, compared to the V4–V5 dataset (the largest difference was in the order *Alteromonadales*, with total of 113 and 49 ASVs, respectively). These taxonomic groups are typically associated with organic matter degradation (Buchan et al., 2014), and were previously shown to dominate sea ice microbial communities associated with algal aggregates (Rapp et al., 2018), as well as surface waters during phytoplankton blooms (Fadeev et al., 2018). Furthermore, the family *Woeseiaceae* (class *Gammaproteobacteria*) also consisted of ca. 30% more ASVs in the V3–V4 dataset, compared to the V4–V5 dataset (total of 127 and 98 ASVs, respectively; Figure 2). This bacterial family is abundant in deep-sea sediments around the globe, including the Arctic Ocean (Bienhold et al., 2016; Hoffmann et al., 2020).

Compared to the V3–V4 dataset, the V4–V5 dataset consisted of at least one third more ASVs in the classes *Phycisphaerae* (total of 206 and 117 ASVs, respectively) and *Planctomycetes* (total of 299 and 244 ASVs, respectively). This difference in the number of observed ASVs was mainly associated with the families *Pirellulaceae* that comprised ca. 2% of all sequences in both datasets (Figure 2), as well as *Phycisphaeraceae* that comprised less than 1% of all sequences (Supplementary Table 3) in both datasets. These taxonomic groups have been previously shown to be associated with sinking particles in the deep ocean and are also abundant in Arctic deep-sea sediments (Fadeev et al., 2020). Furthermore, the archaeal class *Nitrososphaeria* was almost absent from the V3–V4 dataset, with only a few sequences associated with four ASVs, compared to 168 ASVs in the V4–V5 dataset that comprised 7% of the total sequences (Figure 2). Marine members of the *Archaea* in general, and the class *Nitrososphaeria* in particular, are abundant in the Arctic marine environment and can reach up to one fifth of the cells in Arctic microbial communities (Müller et al., 2018; Cardozo-Mino et al., 2020). Taken together, these observations suggest that on ASV level the diversity of different taxonomic groups are captured differently by the two primer sets. This is potentially a result of differences in the regional hypervariability of the 16S rRNA gene within different taxonomic groups (Yang et al., 2016; Kerrigan et al., 2019). In addition, as was previously shown for various taxonomic groups, such as the SAR11 clade, differences in captured diversity may occur also due to specificity differences of the primer sets to the targeted 16S rRNA gene region (Parada et al., 2016).

**FIGURE 3:**
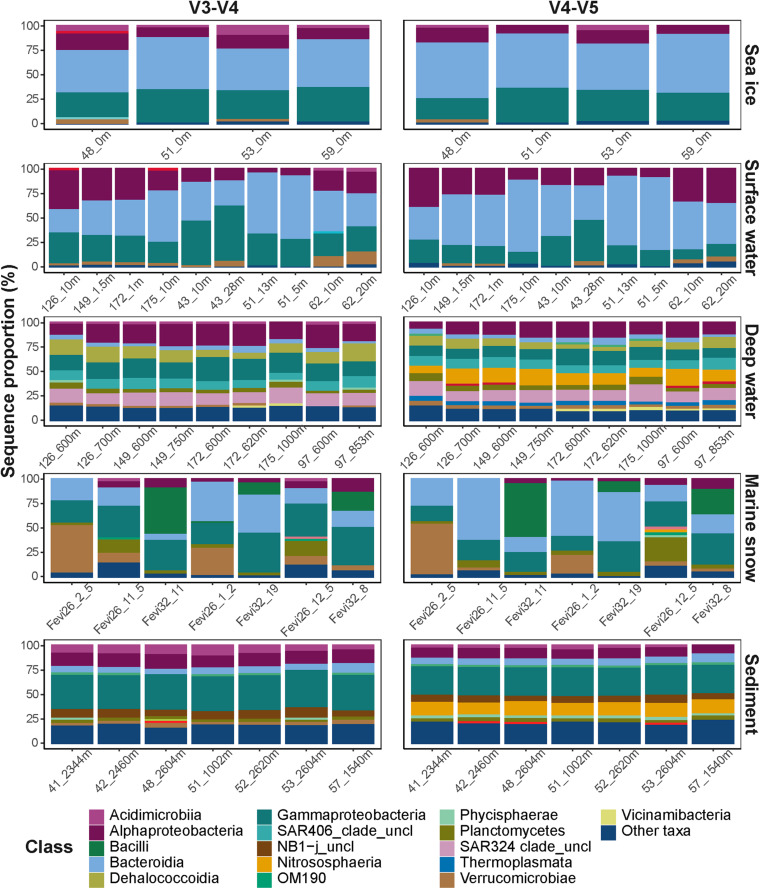
Taxonomic compositions of the microbial communities. Different taxonomic classes are represented by color code. Classes with sequence proportion below 2% were classified as “Other taxa”.

Despite the observed differences on an ASV level, the overall taxonomic composition was consistent between the datasets (Figure 3). Sampled sea ice, surface water, and marine snow communities were dominated by heterotrophic bacteria of the classes *Bacteroidia* (mainly the genus *Polaribacter*) and *Gammaproteobacteria* (mainly the genera in the order *Alteromonadales*), with equivalent relative sequence abundances to those described in previous reports (Bowman et al., 2012; Eronen-Rasimus et al., 2016; Hatam et al., 2016; Wilson et al., 2017; Fadeev et al., 2018, 2020; Rapp et al., 2018). At depth, pelagic communities were dominated by sequences of the class *Alphaproteobacteria*, SAR324 clade, and the archaeal class *Nitrososphaeria*, all of which were previously observed to dominate deep Arctic waters, as well as surface communities during the Arctic winter (Wilson et al., 2017; Fadeev et al., 2020). The sediment communities, which have previously been shown to harbor the highest taxonomic diversity among the described Arctic environments by far (Bienhold et al., 2012; Hoffmann et al., 2017; Rapp et al., 2018), were dominated in sequence abundance of *Gammaproteobacteria*.

**FIGURE 4:**
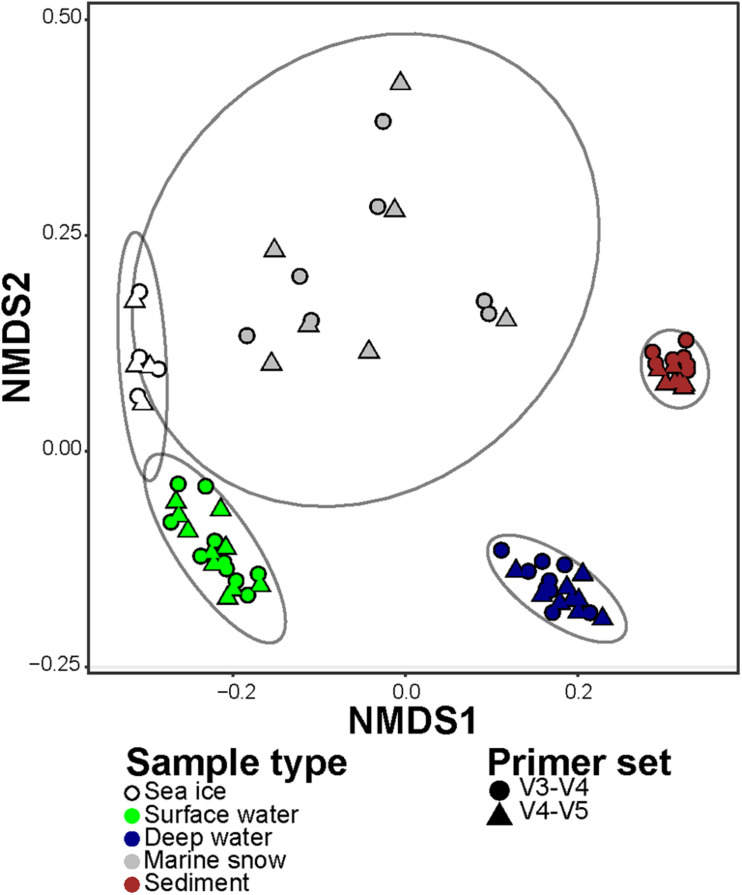
Non-metric Multi-dimensional Scaling (NMDS) of the microbial communities in merged V3–V4 and V4–V5 datasets, based on Jensen-Shannon Divergence. The different types of samples are represented by colors, and the primer set are represented by shapes. Ellipses encompass clustering of each microbiome type with normal confidence of 0.95.

In order to compare the differences in representation of taxonomic groups between the primer sets, we combined sequence abundances of all ASVs according to their taxonomic affiliation at genus rank (i.e., the highest possible shared between the datasets taxonomic resolution). In the V3–V4 dataset, the ASVs were merged into 306 different genera and 279 lineages that were affiliated to higher taxonomic ranks (i.e., were unclassified on a genus rank). In the V4–V5 dataset, the ASVs were merged into 280 different genera and 299 lineages that were affiliated to higher taxonomic ranks. Overall, 489 (72% of the total) lineages were observed in both datasets at this level of taxonomic resolution. In the V3–V4 dataset there were 96 (14% of the total) lineages that were absent from the V4–V5 dataset, but together they comprised less than 1% of the sequences in the V3–V4 dataset. On the other hand, in the V4–V5 dataset there were 90 (13% of the total) lineages that were absent from the V3–V4 dataset, and together they comprised 5% of the sequences in the V4–V5 dataset. In addition, the dissimilarity of community compositions in merged V3–V4 and V4–V5 datasets revealed consistent and significant difference between the microbiomes captured by both primer sets (Two-way PERMANOVA test; *F*_4,64_ = 86.29, *R*^2^ = 0.83, *p* value < 0.001; Figure 4). Only a small fraction of the total variance was associated with the difference between the primer sets (Two-way PERMANOVA test; *F*_1,64_ = 7.59, *R*^2^ = 0.02, *p* value < 0.001). No significant combined effect of different primer sets on different sample types was observed (Two-way PERMANOVA test; *p* value > 0.05). Taken together, these results confirm that, even though the primer sets showed different sensitivity to diversity at the ASV level, both of them reflect similar taxonomic composition down to the genus rank.

The research at the FRAM Microbial Observatory is focused on the seasonal and interannual dynamics of the Arctic Ocean associated with changes in sea ice extent and primary production in the surface ocean (e.g., Metfies et al., 2017; Fadeev et al., 2018, 2020). To further evaluate the performance of the two primer sets in these long-term monitored environments, we compared the sequence representation of selected taxonomic groups, which are associated with distinct stages of seasonal dynamics (Fadeev et al., 2018), to microscopically counted cells using CARD-FISH combined with an automated image acquisition (Cardozo-Mino et al., 2020). Fluorescence *in situ* hybridization techniques have the advantage of providing absolute abundances of (viable) cells that can be directly compared between samples. In microscopy counts of both sea ice and surface water communities the highest observed cell abundance was of the class *Bacteroidia* (up to 35 and 26% of the total microbial community, respectively), which was consistent with the representation of this taxonomic group by both primer sets. In surface water communities, high levels of consistency between the microscopy counts and both primer sets were observed also in the representation of *Alteromonadales* and *Polaribacter* (Table 1). On the other hand, the representation, by both primer sets, of the class *Gammaproteobacteria* in sea ice and surface water communities was 2–4 times higher in comparison to the proportion observed in microscopy counts (up to 9 and 18%, respectively). In contrast, the proportional abundance of the SAR11 clade was 5–10 times higher in the microscopy counts, compared to its representation by both primer sets (Table 1). Our results suggest that at least for some taxonomic groups (i.e., *Polaribacter*), both primer sets may provide a consistent semi-quantitative representation. However, microscopy results must be interpreted under several methodological caveats, knowing that low cellular ribosome content or low efficiency of the probe may alter the representation of individual taxa in our cell counts (Amann and Fuchs, 2008). Therefore, the observed inconsistency in the representation of some taxonomic groups (i.e., SAR11 clade) may also result from these limitations. In order to further investigate the quantitative performance of the primer sets, further investigation, using techniques such as mock communities (Yeh et al., 2019) or metagenomics (McNichol et al., 2020), is required.

**TABLE 1:** Overview of cell abundances and sequence proportions range in selected taxonomic groups.

Sample	Taxonomic group	Abundance (10^5^ cells mL^–1^)	% of DAPI counts	% of total community (V3–V4 dataset)	% of total community (V4–V5 dataset)
Sea ice	*Gammaproteobacteria* (c)	1.0–2.2	8–9%	34–35%	28–35%
	*Alteromonadales* (o)	0.2–0.7	1–6%	23–25%	19–26%
	*Bacteroidia* (c)	2.1–3.9	9–35%	48–52%	55–60%
	*Polaribacter* (g)	1.0–1.5	4–13%	16–17%	14–20%
	SAR11 clade (o)	0.2–0.7	1–6%	0.2–0.4%	0.4–0.8%
Surface water	*Gammaproteobacteria* (c)	4.9–9.8	13–18%	32–55%	19–42%
	*Alteromonadales* (o)	0.8–2.6	2–7%	1–8%	1–4%
	*Bacteroidia* (c)	10.0–12.0	23–26%	25–61%	35–70%
	*Polaribacter* (g)	5.0–7.7	9–20%	11–35%	13–36%
	SAR11 clade (o)	11.5–15.4	29–30%	1–6%	3–10%

*The selected probes and their coverage are described in Supplementary Table 3. c, class; o, order; g, genus.*

## Concluding Remarks

To understand the links between the rapid environmental changes in the Arctic region and the dynamics of microbial communities in the Arctic Ocean, there is a need for robust methods addressing changes in diversity and relative abundance. In order to conduct such observations using a 16S rRNA gene tag-sequencing approach, optimally a similar extraction method and a single PCR primer set should be selected, which can be applied to all environments of the Arctic Ocean (sea ice, water column, and deep-sea sediment). The most suitable primer set for 16S rRNA amplification and sequencing from environmental samples should produce high-quality amplicon libraries and cover with minimum biases the variety of present organisms, as well as their relative abundances. We have found that at all taxonomic ranks down to genus, both primer sets represent the overall richness of the major bacterial taxonomic groups at comparable levels across the different Arctic Ocean biomes. The relative sequence abundance of some dominant taxonomic groups, such as the *Polaribacter*, corresponds with their proportional representation *via* microscopic cell counts. Other taxonomic groups such as the SAR11 clade strongly differ between the molecular and the microscopical representations. However, this discrepancy may be due to limitations of the microscopical quantification. On an ASV level, both primer sets capture the diversity within the most abundant taxonomic groups differently, and thus, the use of each primer set may depend on the target groups. However, the main advantage of the V4–V5 primer set is its additional coverage of the archaeal domain, without compromising the detection of other taxonomic groups. Members of the *Archaea* comprise a substantial fraction of Arctic marine microbial communities, particularly during the dark season and in deep waters. Thus, given the demonstrated similarities and differences, we endorse the use of the V4–V5 primer set for capturing comprehensive insights into microbial community dynamics of the Arctic marine environment.

## Data Availability Statement

The datasets presented in this study can be found in online repositories. The names of the repository/repositories and accession number(s) can be found in the article/Supplementary Material.

## Author Contributions

EF and AB designed the study. CB, IS, MM, and JR provided the environmental samples for the study. HT conducted the sequencing of the samples. MC-M and VS-C conducted the CARD-FISH counts. EF analyzed the data and wrote the manuscript. All authors contributed to the final version of the manuscript.

## Conflict of Interest

The authors declare that the research was conducted in the absence of any commercial or financial relationships that could be construed as a potential conflict of interest.

## Footnotes

1 www.pangaea.de

2 http://www.Rproject.org/

3 http://www.rstudio.com/

4 Biomers.net

## References

[B1] Alonso-Sáez L. Sánchez O. Gasol J. M. Balagué V. Pedrós-Alio C. (2008). Winter-to-summer changes in the composition and single-cell activity of near-surface Arctic prokaryotes. *Environ. Microbiol.* 10 2444–2454. 10.1111/j.1462-2920.2008.01674.x 18557769

[B2] Amann R. Fuchs B. M. (2008). Single-cell identification in microbial communities by improved fluorescence in situ hybridization techniques. *Nat. Rev. Microbiol.* 6 339–348. 10.1038/nrmicro1888 18414500

[B3] Apprill A. McNally S. Parsons R. Weber L. (2015). Minor revision to V4 region SSU rRNA 806R gene primer greatly increases detection of SAR11 bacterioplankton. *Aquat. Microb. Ecol.* 75 129–137. 10.3354/ame01753

[B4] Armougom F. (2009). Exploring microbial diversity using 16S rRNA high-throughput methods. *J. Comput. Sci. Syst. Biol.* 2 74–92. 10.4172/jcsb.1000019

[B5] Avcı B. Krüger K. Fuchs B. M. Teeling H. Amann R. I. (2020). Polysaccharide niche partitioning of distinct Polaribacter clades during North Sea spring algal blooms. *ISME J.* 14 1369–1383. 10.1038/s41396-020-0601-y 32071394 PMC7242417

[B6] Bauerfeind E. Nöthig E.-M. Beszczynska A. Fahl K. Kaleschke L. Kreker K. (2009). Particle sedimentation patterns in the eastern Fram Strait during 2000–2005: results from the Arctic long-term observatory HAUSGARTEN. *Deep Sea Res. Part I Oceanogr. Res. Pap.* 56 1471–1487. 10.1016/j.dsr.2009.04.011

[B7] Bennke C. M. Reintjes G. Schattenhofer M. Ellrott A. Wulf J. Zeder M. (2016). Modification of a high-throughput automatic microbial cell enumeration system for shipboard analyses. *Appl. Environ. Microbiol.* 82 3289–3296. 10.1128/AEM.03931-15 27016562 PMC4959242

[B8] Bienhold C. Boetius A. Ramette A. (2012). The energy–diversity relationship of complex bacterial communities in Arctic deep-sea sediments. *ISME J.* 6 724–732. 10.1038/ismej.2011.140 22071347 PMC3309351

[B9] Bienhold C. Zinger L. Boetius A. Ramette A. (2016). Diversity and biogeography of bathyal and abyssal seafloor bacteria. *PLoS One* 11:e0148016. 10.1371/journal.pone.0148016 26814838 PMC4731391

[B10] Bodungen B. V. Wunsch M. Fürderer H. (2013). “Sampling and analysis of suspended and sinking particles in the Northern North Atlantic,” in *Marine Particles: Analysis and Characterization*, eds Hurd D. C. Spencer D. W., (Washington, DC: American Geophysical Union), 47–56. 10.1029/GM063p0047

[B11] Boetius A. Anesio A. M. Deming J. W. Mikucki J. A. Rapp J. Z. (2015). Microbial ecology of the cryosphere: sea ice and glacial habitats. *Nat. Rev. Microbiol.* 13 677–690. 10.1038/nrmicro3522 26344407

[B12] Bowman J. S. Rasmussen S. Blom N. Deming J. W. Rysgaard S. Sicheritz-Ponten T. (2012). Microbial community structure of Arctic multiyear sea ice and surface seawater by 454 sequencing of the 16S RNA gene. *ISME J.* 6 11–20. 10.1038/ismej.2011.76 21716307 PMC3246233

[B13] Buchan A. LeCleir G. R. Gulvik C. A. Gonzalez J. M. (2014). Master recyclers: features and functions of bacteria associated with phytoplankton blooms. *Nat. Rev. Microbiol.* 12 686–698. 10.1038/nrmicro3326 25134618

[B14] Buttigieg P. L. Fadeev E. Bienhold C. Hehemann L. Offre P. Boetius A. (2018). Marine microbes in 4D — using time series observation to assess the dynamics of the ocean microbiome and its links to ocean health. *Curr. Opin. Microbiol.* 43 169–185. 10.1016/j.mib.2018.01.015 29477022

[B15] Callahan B. J. McMurdie P. J. Rosen M. J. Han A. W. Johnson A. J. A. Holmes S. P. (2016a). DADA2: high-resolution sample inference from Illumina amplicon data. *Nat. Methods* 13 581–583. 10.1038/nmeth.3869 27214047 PMC4927377

[B16] Callahan B. J. Sankaran K. Fukuyama J. A. McMurdie P. J. Holmes S. P. (2016b). Bioconductor Workflow for Microbiome Data Analysis: from raw reads to community analyses. *F1000 Res.* 5:1492. 10.12688/f1000research.8986.2 27508062 PMC4955027

[B17] Caporaso J. G. Lauber C. L. Walters W. A. Berg-Lyons D. Huntley J. Fierer N. (2012). Ultra-high-throughput microbial community analysis on the Illumina HiSeq and MiSeq platforms. *ISME J.* 6 1621–1624. 10.1038/ismej.2012.8 22402401 PMC3400413

[B18] Cardozo-Mino M. G. Fadeev E. Salman-Carvalho V. Boetius A. (2020). Spatial dynamics in Arctic bacterioplankton community densities are strongly linked to distinct physical and biological processes (Fram Strait, 79° N). *bioRxiv[Preprint]* 10.1101/2020.09.02.277574

[B19] Dai A. Luo D. Song M. Liu J. (2019). Arctic amplification is caused by sea-ice loss under increasing CO_2_. *Nat. Commun.* 10:121. 10.1038/s41467-018-07954-9 30631051 PMC6328634

[B20] Diepenbroek M. Glöckner F. O. Grobe P. Güntsch A. Huber R. König-Ries B. (2014). “Towards an integrated biodiversity and ecological research data management and archiving platform: the German federation for the curation of biological data (GFBio),” in *Informatik 2014*, eds Plödereder E. Grunske L. Schneider E. Ull D., (Bonn: Gesellschaft für Informatik e.V), 1711–1721.

[B21] Eronen-Rasimus E. Piiparinen J. Karkman A. Lyra C. Gerland S. Kaartokallio H. (2016). Bacterial communities in Arctic first-year drift ice during the winter/spring transition. *Environ. Microbiol. Rep.* 8 527–535. 10.1111/1758-2229.12428 27264318

[B22] Fadeev E. Rogge A. Ramondenc S. Nöthig E.-M. Wekerle C. Bienhold C. (2020). Sea-ice retreat may decrease carbon export and vertical microbial connectivity in the Eurasian Arctic basins. *Nat. Res.* [Preprint]. 10.21203/rs.3.rs-101878/v1 PMC8566512 34732822

[B23] Fadeev E. Salter I. Schourup-Kristensen V. Nöthig E.-M. Metfies K. Engel A. (2018). Microbial communities in the east and west fram strait during sea ice melting season. *Front. Mar. Sci.* 5:429. 10.3389/fmars.2018.00429

[B24] Fuhrman J. A. Cram J. A. Needham D. M. (2015). Marine microbial community dynamics and their ecological interpretation. *Nat. Rev. Microbiol.* 13 133–146. 10.1038/nrmicro3417 25659323

[B25] Galand P. E. Pereira O. Hochart C. Auguet J. C. Debroas D. (2018). A strong link between marine microbial community composition and function challenges the idea of functional redundancy. *ISME J.* 12 2470–2478. 10.1038/s41396-018-0158-1 29925880 PMC6155072

[B26] Ghiglione J.-F. Galand P. E. Pommier T. Pedrós-Alió C. Maas E. W. Bakker K. (2012). Pole-to-pole biogeography of surface and deep marine bacterial communities. *Proc. Natl. Acad. Sci. U.S.A.* 109 17633–17638. 10.1073/pnas.1208160109 23045668 PMC3491513

[B27] Gilbert J. A. Jansson J. K. Knight R. (2014). The Earth Microbiome project: successes and aspirations. *BMC Biol.* 12:69. 10.1186/s12915-014-0069-1 25184604 PMC4141107

[B28] Gómez-Pereira P. R. Fuchs B. M. Alonso C. Oliver M. J. van Beusekom J. E. E. Amann R. (2010). Distinct flavobacterial communities in contrasting water masses of the North Atlantic Ocean. *ISME J.* 4 472–487. 10.1038/ismej.2009.142 20054356

[B29] Gómez-Rubio V. (2017). *ggplot2 - Elegant Graphics for Data Analysis*, 2nd Edn. 10.18637/jss.v077.b02

[B30] Harrison P. W. Alako B. Amid C. Cerdeño-Tárraga A. Cleland I. Holt S. (2019). The European nucleotide archive in 2018. *Nucleic Acids Res.* 47 D84–D88. 10.1093/nar/gky1078 30395270 PMC6323982

[B31] Hatam I. Lange B. Beckers J. Haas C. Lanoil B. (2016). Bacterial communities from Arctic seasonal sea ice are more compositionally variable than those from multi-year sea ice. *ISME J.* 10 2543–2552. 10.1038/ismej.2016.4 26882269 PMC5030698

[B32] Hoffmann K. Bienhold C. Buttigieg P. L. Knittel K. Laso-Pérez R. Rapp J. Z. (2020). Diversity and metabolism of Woeseiales bacteria, global members of marine sediment communities. *ISME J.* 14 1042–1056. 10.1038/s41396-020-0588-4 31988474 PMC7082342

[B33] Hoffmann K. Hassenrück C. Salman-Carvalho V. Holtappels M. Bienhold C. (2017). Response of bacterial communities to different detritus compositions in arctic deep-sea sediments. *Front. Microbiol.* 8:266. 10.3389/fmicb.2017.00266 28286496 PMC5323390

[B34] Hsieh T. C. Ma K. H. Chao A. (2016). iNEXT: an R package for rarefaction and extrapolation of species diversity (Hill numbers). *Methods Ecol. Evol.* 7 1451–1456. 10.1111/2041-210X.12613

[B35] Karl D. M. Church M. J. (2014). Microbial oceanography and the Hawaii Ocean Time-series programme. *Nat. Rev. Microbiol.* 12 699–713. 10.1038/nrmicro3333 25157695

[B36] Kerrigan Z. Kirkpatrick J. B. D’Hondt S. (2019). Influence of 16S rRNA hypervariable region on estimates of bacterial diversity and community composition in seawater and marine sediment. *Front. Microbiol.* 10:1640. 10.3389/fmicb.2019.01640 31379788 PMC6646839

[B37] Kirchman D. L. Elifantz H. Dittel A. I. Malmstrom R. R. Cottrell M. T. (2007). Standing stocks and activity of archaea and bacteria in the western Arctic Ocean. *Limnol. Oceanogr.* 52 495–507. 10.4319/lo.2007.52.2.0495

[B38] Kirchman D. L. Morán X. A. G. Ducklow H. (2009). Microbial growth in the polar oceans - Role of temperature and potential impact of climate change. *Nat. Rev. Microbiol.* 7 451–459. 10.1038/nrmicro2115 19421189

[B39] Klindworth A. Pruesse E. Schweer T. Peplies J. Quast C. Horn M. (2013). Evaluation of general 16S ribosomal RNA gene PCR primers for classical and next-generation sequencing-based diversity studies. *Nucleic Acids Res.* 41:e1. 10.1093/nar/gks808 22933715 PMC3592464

[B40] Kraemer S. Ramachandran A. Colatriano D. Lovejoy C. Walsh D. A. (2020). Diversity and biogeography of SAR11 bacteria from the Arctic Ocean. *ISME J.* 14 79–90. 10.1038/s41396-019-0499-4 31501503 PMC6908578

[B41] Lalande C. Bauerfeind E. Nöthig E. Beszczynska-Möller A. (2013). Impact of a warm anomaly on export fluxes of biogenic matter in the eastern Fram Strait. *Prog. Oceanogr.* 109 70–77. 10.1016/j.pocean.2012.09.006

[B42] Lenton T. M. Rockström J. Gaffney O. Rahmstorf S. Richardson K. Steffen W. (2019). Climate tipping points — too risky to bet against. *Nature* 575 592–595. 10.1038/d41586-019-03595-0 31776487

[B43] Liu Y. Blain S. Crispi O. Rembauville M. Obernosterer I. (2020). Seasonal dynamics of prokaryotes and their associations with diatoms in the Southern Ocean as revealed by an autonomous sampler. *Environ. Microbiol.* 22 3968–3984. 10.1111/1462-2920.15184 32755055

[B44] Martin M. (2011). Cutadapt removes adapter sequences from high-throughput sequencing reads. *EMBnet J.* 17:10. 10.14806/ej.17.1.200

[B45] McMurdie P. J. Holmes S. (2013). phyloseq: an R package for reproducible interactive analysis and graphics of microbiome census data. *PLoS One* 8:e61217. 10.1371/journal.pone.0061217 23630581 PMC3632530

[B46] McNichol J. C. Berube P. M. Biller S. J. Fuhrman J. A. (2020). Evaluating and improving SSU rRNA PCR primer coverage via metagenomes from global ocean surveys. *bioRxiv [Preprint]* 10.1101/2020.11.09.375543 PMC8269242 34060911

[B47] Metfies K. Bauerfeind E. Wolf C. Sprong P. Frickenhaus S. Kaleschke L. (2017). Protist communities in moored long-term sediment traps (Fram Strait, Arctic)–preservation with mercury chloride allows for PCR-based molecular genetic analyses. *Front. Mar. Sci.* 4:301. 10.3389/fmars.2017.00301

[B48] Müller O. Wilson B. Paulsen M. L. Ruminska A. Armo H. R. Bratbak G. (2018). Spatiotemporal dynamics of ammonia-oxidizing Thaumarchaeota in Distinct Arctic water masses. *Front. Microbiol.* 9:24. 10.3389/fmicb.2018.00024 29410658 PMC5787140

[B49] Nikrad M. P. Cottrell M. T. Kirchman D. L. (2012). Abundance and single-cell activity of heterotrophic bacterial groups in the Western Arctic Ocean in summer and winter. *Appl. Environ. Microbiol.* 78 2402–2409. 10.1128/AEM.07130-11 22286998 PMC3302604

[B50] Oksanen J. Kindt R. Legendre P. O’Hara B. Stevens M. H. H. Oksanen M. J. (2007). The vegan package. *Commun. Ecol. Packag.* 10:719.

[B51] Parada A. E. Needham D. M. Fuhrman J. A. (2016). Every base matters: assessing small subunit rRNA primers for marine microbiomes with mock communities, time series and global field samples. *Environ. Microbiol.* 18 1403–1414. 10.1111/1462-2920.13023 26271760

[B52] Pedrós-Alió C. Potvin M. Lovejoy C. (2015). Diversity of planktonic microorganisms in the Arctic Ocean. *Prog. Oceanogr.* 139 233–243. 10.1016/j.pocean.2015.07.009

[B53] Peeken I. Primpke S. Beyer B. Gütermann J. Katlein C. Krumpen T. (2018). Arctic sea ice is an important temporal sink and means of transport for microplastic. *Nat. Commun.* 9:1505. 10.1038/s41467-018-03825-5 29692405 PMC5915590

[B54] Peng G. Meier W. N. (2018). Temporal and regional variability of Arctic sea-ice coverage from satellite data. *Ann. Glaciol.* 59 191–200. 10.1017/aog.2017.32

[B55] Pernthaler A. Pernthaler J. Amann R. (2002). Fluorescence in situ hybridization and catalyzed reporter deposition for the identification of marine bacteria. *Appl. Environ. Microbiol.* 68 3094–3101. 10.1128/AEM.68.6.3094-3101.2002 12039771 PMC123953

[B56] Quast C. Pruesse E. Yilmaz P. Gerken J. Schweer T. Glo F. O. (2013). The SILVA ribosomal RNA gene database project : improved data processing and web-based tools. *Nucleic Acids Res.* 41 D590–D596. 10.1093/nar/gks1219 23193283 PMC3531112

[B57] Rapp J. Z. Fernández-Méndez M. Bienhold C. Boetius A. (2018). Effects of ice-algal aggregate export on the connectivity of bacterial communities in the central Arctic Ocean. *Front. Microbiol.* 9:1035. 10.3389/fmicb.2018.01035 29875749 PMC5974969

[B58] Royo-Llonch M. Sánchez P. Ruiz-González C. Salazar G. Pedrós-Alió C. Labadie K. (2020). Ecogenomics of key prokaryotes in the arctic ocean. *bioRxiv [Preprint]* 10.1101/2020.06.19.156794

[B59] Schlitzer R. (2018). *Ocean Data View.* Available online at: https://odv.awi.de (accessed July 28, 2019).

[B60] Soltwedel T. Bauerfeind E. Bergmann M. Bracher A. Budaeva N. Busch K. (2015). Natural variability or anthropogenically-induced variation? Insights from 15 years of multidisciplinary observations at the arctic marine LTER site HAUSGARTEN. *Ecol. Indic.* 65 89–102. 10.1016/j.ecolind.2015.10.001

[B61] Soltwedel T. Bauerfeind E. Bergmann M. Budaeva N. Hoste E. Jaeckisch N. (2005). HAUSGARTEN: multidisciplinary investigations at a deep-sea, long-term observatory in the Arctic Ocean. *Oceanography* 18 46–61. 10.5670/oceanog.2005.24

[B62] Thomas T. Gilbert J. Meyer F. (2012). Metagenomics - a guide from sampling to data analysis. *Microb. Inform. Exp.* 2:3. 10.1186/2042-5783-2-3 22587947 PMC3351745

[B63] Wang Y. Qian P.-Y. (2009). Conservative fragments in bacterial 16S rRNA genes and primer design for 16S Ribosomal DNA amplicons in metagenomic studies. *PLoS One* 4:e7401. 10.1371/journal.pone.0007401 19816594 PMC2754607

[B64] Wassmann P. Duarte C. M. Agustí S. Sejr M. K. (2011). Footprints of climate change in the Arctic marine ecosystem. *Glob. Chang. Biol.* 17 1235–1249. 10.1111/j.1365-2486.2010.02311.x

[B65] Wassmann P. Reigstad M. (2011). Future Arctic Ocean seasonal ice zones and implications for pelagic-benthic coupling. *Oceanography* 24 220–231. 10.5670/oceanog.2011.74

[B66] Wear E. K. Wilbanks E. G. Nelson C. E. Carlson C. A. (2018). Primer selection impacts specific population abundances but not community dynamics in a monthly time-series 16S rRNA gene amplicon analysis of coastal marine bacterioplankton. *Environ. Microbiol.* 20 2709–2726. 10.1111/1462-2920.14091 29521439 PMC6175402

[B67] Willis C. Desai D. Laroche J. (2019). Influence of 16S rRNA variable region on perceived diversity of marine microbial communities of the Northern North Atlantic. *FEMS Microbiol. Lett.* 366:fnz152. 10.1093/femsle/fnz152 31344223 PMC6673769

[B68] Wilson B. Müller O. Nordmann E. L. Seuthe L. Bratbak G. Øvreås L. (2017). Changes in marine prokaryote composition with season and depth over an Arctic polar year. *Front. Mar. Sci.* 4:95. 10.3389/fmars.2017.00095

[B69] Yang B. Wang Y. Qian P.-Y. (2016). Sensitivity and correlation of hypervariable regions in 16S rRNA genes in phylogenetic analysis. *BMC Bioinform.* 17:135. 10.1186/s12859-016-0992-y 27000765 PMC4802574

[B70] Yeh Y.-C. McNichol J. Needham D. M. Fichot E. B. Fuhrman J. A. (2019). Comprehensive single-PCR 16S and 18S rRNA community analysis validated with mock communities and denoising algorithms. *bioRxiv [Preprint]* 10.1101/866731 33938123

[B71] Yilmaz P. Parfrey L. W. Yarza P. Gerken J. Pruesse E. Quast C. (2014). The SILVA and “All-species Living Tree Project (LTP)” taxonomic frameworks. *Nucleic Acids Res.* 42 D643–D648. 10.1093/nar/gkt1209 24293649 PMC3965112

[B72] Zeder M. Ellrott A. Amann R. (2011). Automated sample area definition for high−throughput microscopy. *Cytom. Part A* 79 306–310. 10.1002/cyto.a.21034 21412981

