# Supplementary material for: Comparison of Two 16S rRNA Primers (V3–V4 and V4–V5) for Studies of Arctic Microbial Communities
Source: Front Microbiol. 2021 Feb 16;12:637526. doi: 10.3389/fmicb.2021.637526 (PMC7920977; doi:10.3389/fmicb.2021.637526)
Supplement: Supplementary file 1 [file Data_Sheet_1.PDF]

Supplementary information

Comparison of two 16S rRNA primers (V3-V4 and V4-V5) for studies of Arctic microbial communities

Eduard Fadeev, Magda G Cardozo-Mino, Josephine Z Rapp, Christina Bienhold, Ian Salter, Verena Salman-Carvalho, Massimiliano Molari, Halina E. Tegetmeyer, Pier Luigi Buttigieg, Antje Boetius

*** Correspondence:**
Eduard Fadeev
dr.eduard.fadeev@gmail.com

Figures and Tables

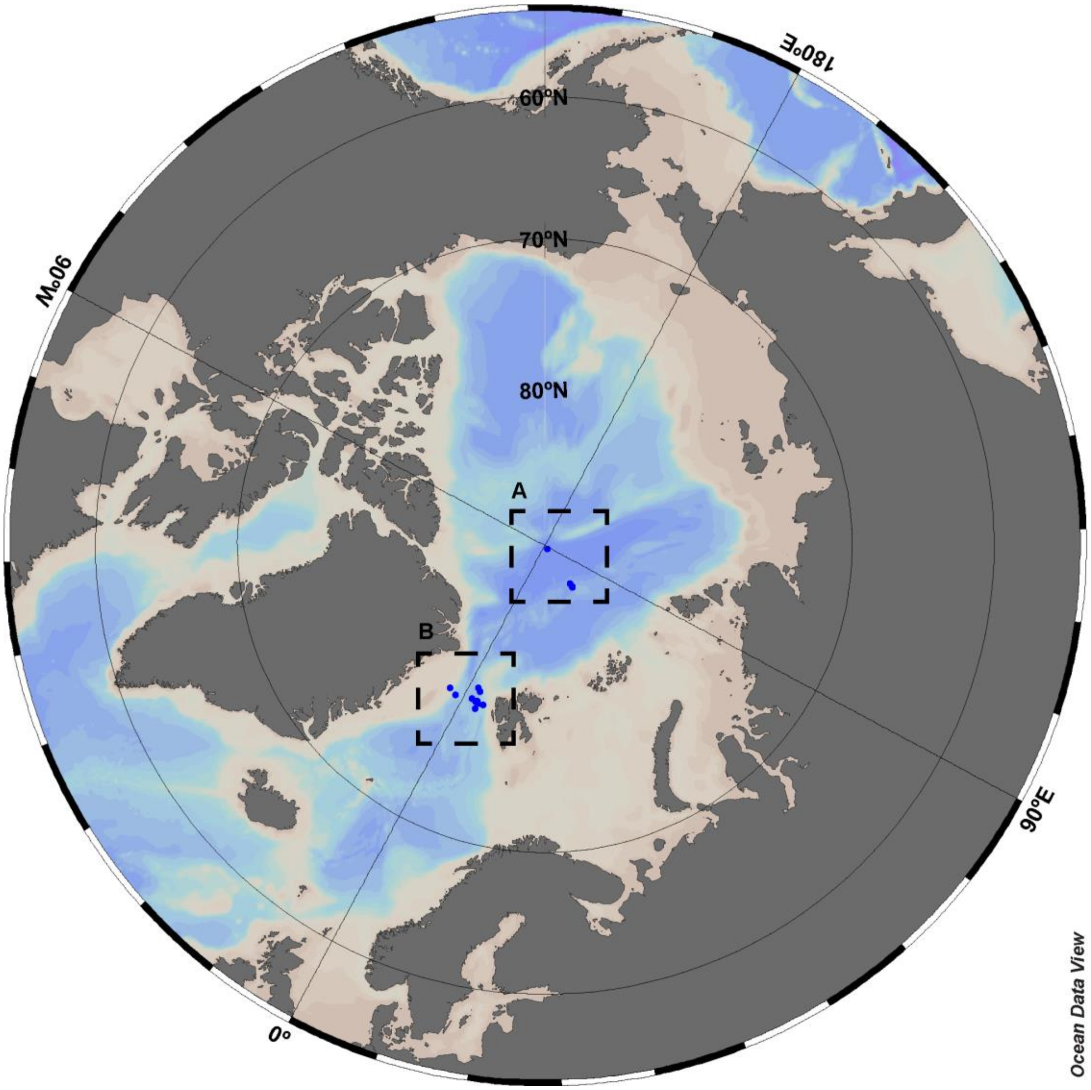

Supplementary Figure 1. Overview of sampling locations. Panel A marks the central Arctic Ocean where surface and deep ocean samples were collected. Panel B marks the Fram Strait (in the Greenland Sea) where sea ice, surface and deep water, marine snow and sediment samples were collected. The figure was produced using Ocean Data View.

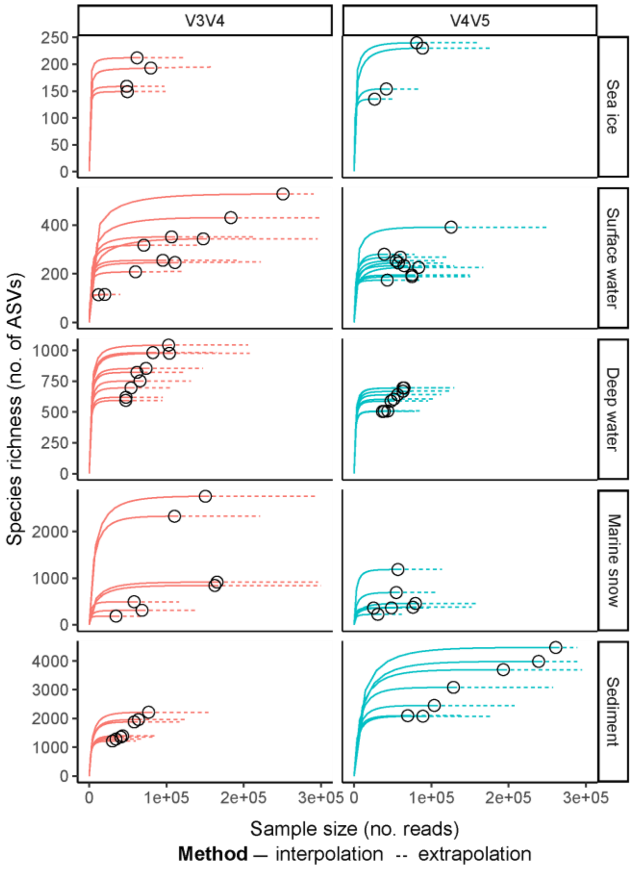

Supplementary Figure 2. Rarefactions of 16S rRNA gene analysis of bacterial and archaeal communities in both V3-V4 and V4-V5 datasets. The solid lines represent the observed accumulation with the number of reads sampled, and the dashed lines represent the extrapolated accumulation up to the double amount of reads. The observed values for each community are denoted by solid shapes. Sample-size-based rarefaction curves generated with the R-package "iNEXT", based on the Hill number of order q = 0. The rarefaction curves for each sample were generated based on 40 equally spaced rarefied sample sizes with 100 iterations.

Supplementary Table 1. Overview of microbial samples included in the study.

Sample Name	PANGAEA event ID	Sample type (sampling device)	Sampling date	Depth (m)	Lat. [ºN]/Long. [ºE]	Geographic region
PS99/48-Sea-ice-depth_0	PS99/48	Sea ice (ice corer)	30/06/2016	0	78.82/-2.73	Greenland Sea
PS99/51-Sea-ice-depth_0	PS99/51-7	Sea ice (ice corer)	02/07/2016	0	78.99/-5.42	Greenland Sea
PS99/53-Sea-ice-depth_0	PS99/53	Sea ice (ice corer)	03/07/2016	0	79.92/3.06	Greenland Sea
PS99/59-Sea-ice-depth_0	PS99/59	Sea ice (ice corer)	06/07/2016	0	79.13/2.84	Greenland Sea
PS101/172-Surface-water-depth_1	PS101/172-20	Surface water (niskin bottle)	01/10/2016	1	86.86/61.58	Arctic Ocean
PS101/175-Surface-water-depth_10	PS101/175-21	Surface water (niskin bottle)	02/10/2016	10	87.10/61.61	Arctic Ocean
PS101/126-Surface-water-depth_10	PS101/126-10	Surface water (niskin bottle)	23/09/2016	10	89.74/61.87	Arctic Ocean
PS101/149-Surface-water-depth_1.5	PS101/149-16	Surface water (niskin bottle)	27/09/2016	1.5	86.79/61.83	Arctic Ocean
PS99/43-Surface-water-depth_10	PS99/43-3	Surface water (niskin bottle)	27/06/2016	10	79.07/4.19	Greenland Sea
PS99/43-Surface-water-depth_28	PS99/43-3	Surface water (niskin bottle)	27/06/2016	28	79.07/4.19	Greenland Sea
PS99/51-Surface-water-depth_5	PS99/51-2	Surface water (niskin bottle)	02/07/2016	5	78.99/-5.42	Greenland Sea
PS99/51-Surface-water-depth_13	PS99/51-2	Surface water (niskin bottle)	02/07/2016	13	78.99/-5.42	Greenland Sea
PS99/62-Surface-water-depth_10	PS99/62-4	Surface water (niskin bottle)	08/07/2016	10	79.03/7.00	Greenland Sea

Sample Name	PANGAEA event ID	Sample type (sampling device)	Sampling date	Depth (m)	Lat. [ºN]/Long. [ºE]	Geographic region
PS99/62-Surface-water-depth_20	PS99/62-4	Surface water (niskin bottle)	08/07/2016	20	79.03/7.00	Greenland Sea
PS101/149-Deep-water-depth_600	PS101/149-16	Deep ocean (niskin bottle)	27/09/2016	600	86.79/61.83	Arctic Ocean
PS101/149-Deep-water-depth_750	PS101/149-16	Deep ocean (niskin bottle)	27/09/2016	750	86.79/61.83	Arctic Ocean
PS101/97-Deep-water-depth_600	PS101/97-6	Deep ocean (niskin bottle)	19/09/2016	600	86.82/61.81	Arctic Ocean
PS101/97-Deep-water-depth_853	PS101/97-6	Deep ocean (niskin bottle)	19/09/2016	853	86.82/61.81	Arctic Ocean
PS101/172-Deep-water-depth_600	PS101/172-20	Deep ocean (niskin bottle)	01/10/2016	600	86.86/61.58	Arctic Ocean
PS101/172-Deep-water-depth_620	PS101/172-20	Deep ocean (niskin bottle)	01/10/2016	620	86.86/61.58	Arctic Ocean
PS101/175-Deep-water-depth_1000	PS101/175-21	Deep ocean (niskin bottle)	02/10/2016	1000	87.10/61.61	Arctic Ocean
PS101/126-Deep-water-depth_600	PS101/126-10	Deep ocean (niskin bottle)	23/09/2016	600	89.74/61.87	Arctic Ocean
PS101/126-Deep-water-depth_1000	PS101/126-10	Deep ocean (niskin bottle)	23/09/2016	700	89.74/61.87	Arctic Ocean
FRAM_2987_Fevi_32_oben_8	FEVI32	Marine snow (sediment trap)	31/10/2015 - 30/11/2015	205	79.01/4.33	Greenland Sea
FRAM_2999_Fevi_32_oben_11	FEVI32	Marine snow (sediment trap)	31/01/2016 - 29/02/2016	205	79.01/4.33	Greenland Sea
FRAM_3031_Fevi_32_oben_19	FEVI32	Marine snow (sediment trap)	31/05/2016 - 31/06/2016	205	79.01/4.33	Greenland Sea

Sample Name	PANGAEA event ID	Sample type (sampling device)	Sampling date	Depth (m)	Lat. [ºN]/Long. [ºE]	Geographic region
FRAM_3435_Fevi_26_unten_1_5	FEVI26	Marine snow (sediment trap)	29/07/2012 - 15/08/2012	2356	79.01/4.33	Greenland Sea
FRAM_3443_Fevi_26_unten_2_5	FEVI26	Marine snow (sediment trap)	15/08/2012 - 30/08/2012	2356	79.01/4.33	Greenland Sea
FRAM_3459_Fevi_26_unten_11_5	FEVI26	Marine snow (sediment trap)	31/01/2013 - 28/02/2013	2356	79.01/4.33	Greenland Sea
FRAM_3467_Fevi_26_unten_12_5	FEVI26	Marine snow (sediment trap)	28/02/2013 - 31/03/2013	2356	79.01/4.33	Greenland Sea
PS99/48-Sediment-depth_5000	PS99/48-12	Sediment (multicorer)	30/06/2016	2604	78.82/-2.73	Greenland Sea
PS99/51-Sediment-depth_5000	PS99/51-9	Sediment (multicorer)	02/07/2016	1002	78.99/-5.418	Greenland Sea
PS99/41-Sediment-depth_5000	PS99/41-9	Sediment (multicorer)	25/06/2016	2344	78.61/5.05	Greenland Sea
PS99/42-Sediment-depth_5000	PS99/42-12	Sediment (multicorer)	27/06/2016	2460	79.06/4.19	Greenland Sea
PS99/52-Sediment-depth_5000	PS99/52-3	Sediment (multicorer)	03/07/2016	2620	79.74/4.43	Greenland Sea
PS99/53-Sediment-depth_5000	PS99/53-7	Sediment (multicorer)	03/07/2016	2604	79.92/3.06	Greenland Sea
PS99/57-Sediment-depth_5000	PS99/57-2	Sediment (multicorer)	06/07/2016	1540	79.13/4.9	Greenland Sea

Supplementary Table 2. Characteristics of sequenced libraries. The table consists of sampling information for each sample, number of sequences and calculated alpha diversity indices. (%) represents the proportion of raw sequences.

Sample Name	Sample type	Primer set	Raw sequences	Sequences clustered to ASVs	Tax. filtered sequences	Final sequences	No. of ASVs	Chao1 richness	Shannon diversity index (H)	Inverse Simpson's div. index
PS99/48-Sea-ice-depth_0	Sea ice	V4V5	171765	133494 (78%)	44659 (26%)	88615 (52%)	230	230.0	3.7	11.9
PS99/48-Sea-ice-depth_0	Sea ice	V3V4	203532	98200 (48%)	18318 (9%)	79758 (39%)	193	196.0	3.8	16.0
PS99/51-Sea-ice-depth_0	Sea ice	V4V5	147296	114331 (78%)	72175 (49%)	41684 (28%)	154	154.0	3.7	22.6
PS99/51-Sea-ice-depth_0	Sea ice	V3V4	168177	83660 (50%)	35317 (21%)	48591 (29%)	159	159.0	4.0	30.8
PS99/53-Sea-ice-depth_0	Sea ice	V4V5	166506	128562 (77%)	48287 (29%)	80945 (49%)	240	240.0	4.3	44.6
PS99/53-Sea-ice-depth_0	Sea ice	V3V4	157081	78510 (50%)	17279 (11%)	61932 (39%)	212	212.0	4.5	55.2
PS99/59-Sea-ice-depth_0	Sea ice	V4V5	87520	60338 (69%)	33258 (38%)	26596 (30%)	135	135.0	3.7	23.1
PS99/59-Sea-ice-depth_0	Sea ice	V3V4	148701	75193 (51%)	25279 (17%)	49382 (33%)	149	149.0	3.9	27.2
PS101/126-Surface-water-depth_10	Surface water	V4V5	72239	44892 (62%)	2167 (3%)	42901 (59%)	174	174.0	3.7	16.3
PS101/126-Surface-water-depth_10	Surface water	V3V4	157175	96258 (61%)	1572 (1%)	95260 (61%)	255	255.0	4.0	19.2
PS101/149-Surface-water-depth_1.5	Surface water	V4V5	113152	76345 (67%)	1132 (1%)	74867 (66%)	188	188.0	3.5	14.5
PS101/149-Surface-water-depth_1.5	Surface water	V3V4	180309	111931 (62%)	0 (0%)	111081 (62%)	246	247.5	3.8	17.6
PS101/172-Surface-water-depth_1	Surface water	V4V5	112069	75628 (67%)	1121 (1%)	74639 (67%)	195	195.0	3.5	13.1

**Sample Name**	**Sample type**	**Primer set**	**Raw sequences**	**Sequences clustered to ASVs**	**Tax. filtered sequences**	**Final sequences**	**No. of ASVs**	**Chao1 richness**	**Shannon diversity index (H)**	**Inverse Simpson's div. index**
PS101/172-Surface-water-depth_1	Surface water	V3V4	91905	60150 (65%)	0 (0%)	59949 (65%)	208	208.3	3.8	16.9
PS101/175-Surface-water-depth_10	Surface water	V4V5	99011	66115 (67%)	990 (1%)	64664 (65%)	233	233.0	3.2	8.9
PS101/175-Surface-water-depth_10	Surface water	V3V4	116509	71492 (61%)	1165 (1%)	70774 (61%)	317	317.3	3.8	15.0
PS99/43-Surface-water-depth_10	Surface water	V4V5	176065	145842 (83%)	59862 (34%)	83582 (47%)	226	226.0	3.3	12.2
PS99/43-Surface-water-depth_10	Surface water	V3V4	203212	183230 (90%)	36578 (18%)	147622 (73%)	343	343.5	3.6	14.5
PS99/43-Surface-water-depth_28	Surface water	V4V5	207043	163777 (79%)	107662 (52%)	54262 (26%)	253	253.0	3.7	19.3
PS99/43-Surface-water-depth_28	Surface water	V3V4	176436	160202 (91%)	52931 (30%)	106583 (60%)	351	351.0	3.9	20.4
PS99/51-Surface-water-depth_13	Surface water	V4V5	138385	103567 (75%)	63657 (46%)	38994 (28%)	280	280.0	3.4	10.0
PS99/51-Surface-water-depth_13	Surface water	V3V4	40614	18996 (47%)	6904 (17%)	12018 (30%)	114	114.0	3.2	10.2
PS99/51-Surface-water-depth_5	Surface water	V4V5	127645	80462 (63%)	22976 (18%)	57419 (45%)	244	244.0	3.1	7.4
PS99/51-Surface-water-depth_5	Surface water	V3V4	49588	22779 (46%)	2479 (5%)	20129 (41%)	115	115.0	3.0	8.1
PS99/62-Surface-water-depth_10	Surface water	V4V5	130662	97708 (75%)	37892 (29%)	59753 (46%)	268	268.0	4.1	26.7
PS99/62-Surface-water-depth_10	Surface water	V3V4	242835	219481 (90%)	36425 (15%)	183404 (76%)	430	430.0	4.3	36.6
PS99/62-Surface-water-depth_20	Surface water	V4V5	202440	164608 (81%)	38464 (19%)	125567 (62%)	391	391.0	4.4	38.1

Sample Name	Sample type	Primer set	Raw sequences	Sequences clustered to ASVs	Tax. filtered sequences	Final sequences	No. of ASVs	Chao1 richness	Shannon diversity index (H)	Inverse Simpson's div. index
PS99/62-Surface-water-depth_20	Surface water	V3V4	304914	275111 (90%)	24393 (8%)	250766 (82%)	527	527.4	4.5	45.9
PS101/126-Deep-water-depth_1000	Deep ocean	V4V5	96316	52030 (54%)	0 (0%)	51431 (53%)	604	604.0	5.4	83.8
PS101/126-Deep-water-depth_1000	Deep ocean	V3V4	105208	62440 (59%)	1052 (1%)	61768 (59%)	821	821.0	5.8	100.4
PS101/126-Deep-water-depth_600	Deep ocean	V4V5	110859	65091 (59%)	0 (0%)	64623 (58%)	695	695.2	5.4	60.4
PS101/126-Deep-water-depth_600	Deep ocean	V3V4	123536	74382 (60%)	1235 (1%)	73575 (60%)	853	853.0	5.7	80.9
PS101/149-Deep-water-depth_600	Deep ocean	V4V5	113055	63804 (56%)	0 (0%)	63344 (56%)	695	696.5	5.4	71.3
PS101/149-Deep-water-depth_600	Deep ocean	V3V4	91852	54543 (59%)	0 (0%)	54102 (59%)	695	695.0	5.5	73.5
PS101/149-Deep-water-depth_750	Deep ocean	V4V5	90230	48137 (53%)	0 (0%)	47718 (53%)	588	588.0	5.3	59.3
PS101/149-Deep-water-depth_750	Deep ocean	V3V4	137247	83195 (61%)	0 (0%)	82463 (60%)	981	981.1	5.7	77.3
PS101/172-Deep-water-depth_600	Deep ocean	V4V5	102200	56602 (55%)	0 (0%)	56245 (55%)	640	640.0	5.4	73.0
PS101/172-Deep-water-depth_600	Deep ocean	V3V4	175332	104401 (60%)	0 (0%)	103772 (59%)	976	976.3	5.7	82.1
PS101/172-Deep-water-depth_620	Deep ocean	V4V5	65680	36842 (56%)	0 (0%)	36623 (56%)	504	505.0	5.3	74.9
PS101/172-Deep-water-depth_620	Deep ocean	V3V4	172641	103378 (60%)	0 (0%)	102751 (60%)	1042	1042.5	5.9	102.4
PS101/175-Deep-water-depth_1000	Deep ocean	V4V5	69065	39187 (57%)	0 (0%)	38976 (56%)	505	505.3	5.1	42.7

**Sample Name**	**Sample type**	**Primer set**	**Raw sequences**	**Sequences clustered to ASVs**	**Tax. filtered sequences**	**Final sequences**	**No. of ASVs**	**Chao1 richness**	**Shannon diversity index (H)**	**Inverse Simpson's div. index**
PS101/175-Deep-water-depth_1000	Deep ocean	V3V4	111497	66243 (59%)	0 (0%)	65802 (59%)	752	752.0	5.5	59.0
PS101/97-Deep-water-depth_600	Deep ocean	V4V5	114799	64035 (56%)	0 (0%)	63283 (55%)	668	668.5	5.4	72.0
PS101/97-Deep-water-depth_600	Deep ocean	V3V4	85960	47923 (56%)	0 (0%)	47386 (55%)	594	594.0	5.4	72.5
PS101/97-Deep-water-depth_853	Deep ocean	V4V5	85792	44017 (51%)	0 (0%)	43583 (51%)	508	508.0	5.3	67.2
PS101/97-Deep-water-depth_853	Deep ocean	V3V4	85965	48201 (56%)	0 (0%)	47633 (55%)	619	619.0	5.5	76.8
FRAM_2987_Fevi_32_oben_8	Marine snow	V4V5	76579	55212 (72%)	766 (1%)	54576 (71%)	692	692.0	4.5	18.6
FRAM_2987_Fevi_32_oben_8	Marine snow	V3V4	98381	58701 (60%)	984 (1%)	58134 (59%)	494	494.0	4.5	22.9
FRAM_2999_Fevi_32_oben_11	Marine snow	V4V5	96625	79642 (82%)	0 (0%)	79449 (82%)	458	458.0	2.8	4.5
FRAM_2999_Fevi_32_oben_11	Marine snow	V3V4	118201	68657 (58%)	0 (0%)	68402 (58%)	311	311.0	3.4	8.7
FRAM_3031_Fevi_32_oben_19	Marine snow	V4V5	103508	79945 (77%)	3105 (3%)	76110 (74%)	376	376.0	3.7	12.0
FRAM_3031_Fevi_32_oben_19	Marine snow	V3V4	68227	36464 (53%)	2047 (3%)	34710 (51%)	186	186.0	3.7	13.9
FRAM_3435_Fevi_26_unten_1_5	Marine snow	V4V5	65137	50669 (78%)	1954 (3%)	48342 (74%)	363	363.0	3.9	16.2
FRAM_3435_Fevi_26_unten_1_5	Marine snow	V3V4	206197	172422 (84%)	6186 (3%)	165454 (80%)	916	916.1	4.1	17.5
FRAM_3443_Fevi_26_unten_2_5	Marine snow	V4V5	41562	31646 (76%)	831 (2%)	30775 (74%)	224	224.0	3.1	5.8

Sample Name	Sample type	Primer set	Raw sequences	Sequences clustered to ASVs	Tax. filtered sequences	Final sequences	No. of ASVs	Chao1 richness	Shannon diversity index (H)	Inverse Simpson's div. index
FRAM_3443_Fevi_26_unten_2_5	Marine snow	V3V4	198754	166859 (84%)	3975 (2%)	162576 (82%)	844	844.0	3.5	6.9
FRAM_3459_Fevi_26_unten_11_5	Marine snow	V4V5	42799	29151 (68%)	3852 (9%)	25222 (59%)	360	360.0	4.3	24.0
FRAM_3459_Fevi_26_unten_11_5	Marine snow	V3V4	142545	111866 (78%)	1425 (1%)	110475 (78%)	2329	2329.1	6.2	48.5
FRAM_3467_Fevi_26_unten_12_5	Marine snow	V4V5	87606	58062 (66%)	876 (1%)	56719 (65%)	1187	1187.3	6.0	94.8
FRAM_3467_Fevi_26_unten_12_5	Marine snow	V3V4	193984	153004 (79%)	1940 (1%)	150392 (78%)	2758	2758.8	6.2	41.7
PS99/41-Sediment-depth_5000	Sediment	V4V5	277631	194826 (70%)	0 (0%)	193178 (70%)	3697	3697.1	7.2	434.7
PS99/41-Sediment-depth_5000	Sediment	V3V4	127331	77463 (61%)	0 (0%)	76941 (60%)	2217	2218.2	6.9	450.4
PS99/42-Sediment-depth_5000	Sediment	V4V5	365571	263190 (72%)	3656 (1%)	260989 (71%)	4468	4468.9	7.3	493.5
PS99/42-Sediment-depth_5000	Sediment	V3V4	81997	41072 (50%)	0 (0%)	40874 (50%)	1356	1356.0	6.6	356.3
PS99/48-Sediment-depth_5000	Sediment	V4V5	195846	130331 (67%)	1958 (1%)	128624 (66%)	3082	3082.1	7.1	424.8
PS99/48-Sediment-depth_5000	Sediment	V3V4	108202	58711 (54%)	0 (0%)	58276 (54%)	1879	1879.1	6.8	367.8
PS99/51-Sediment-depth_5000	Sediment	V4V5	334946	241281 (72%)	0 (0%)	238764 (71%)	3985	3985.5	7.1	408.7
PS99/51-Sediment-depth_5000	Sediment	V3V4	74634	43676 (59%)	0 (0%)	43354 (58%)	1395	1395.1	6.5	306.2
PS99/52-Sediment-depth_5000	Sediment	V4V5	151719	104553 (69%)	0 (0%)	103786 (68%)	2453	2453.3	7.0	447.7

Sample Name	Sample type	Primer set	Raw sequences	Sequences clustered to ASVs	Tax. filtered sequences	Final sequences	No. of ASVs	Chao1 richness	Shannon diversity index (H)	Inverse Simpson's div. index
PS99/52-Sediment-depth_5000	Sediment	V3V4	62866	34927 (56%)	0 (0%)	34663 (55%)	1283	1283.3	6.6	370.9

Supplementary Table 3. Overview of ASV composition of bacterial families in both datasets. The table consists of all bacterial families that were observed in both V3-V4 and V4-V5 datasets, and had sequence proportion of at least 1%.

**Class**	**Family**	**Seq. proportion (% of total V3-V4)**	**Seq. proportion (% of total V4-V5)**	**Num. ASVs (V3-V4)**	**Num. ASVs (V4-V5)**	**ASVs ratio (V3-V4/V4-V5)**
Bacteroidia	Flavobacteriaceae	18	18	278	196	1.42
Verrucomicrobiae	Rubritaleaceae	6	1	72	37	1.95
Alphaproteobacteria	Clade_I	5	4	48	49	0.98
Alphaproteobacteria	Rhodobacteraceae	5	3	108	95	1.14
Gammaproteobacteria	Nitrincolaceae	4	2	31	19	1.63
SAR324_clade(Marine_group_B)_uncl	SAR324_clade_uncl	3	2	26	54	0.48
Gammaproteobacteria	Woeseiaceae	2	4	127	98	1.30
Bacteroidia	Cryomorphaceae	2	2	83	76	1.09
Bacteroidia	NS9_marine_group	2	2	55	42	1.31
Dehalococcoidia	SAR202_clade_uncl	2	2	137	162	0.85
Marinimicrobia_(SAR406_clade)_uncl	Marinimicrobia_uncl	2	2	90	93	0.97
Planctomycetes	Pirellulaceae	2	2	205	285	0.72
Gammaproteobacteria	Colwelliaceae	2	1	40	21	1.90
Gammaproteobacteria	Porticoccaceae	2	1	39	21	1.86
Gammaproteobacteria	SAR86_clade_uncl	2	1	35	24	1.46
Verrucomicrobiae	Puniceicoccaceae	2	1	31	35	0.89

Class	Family	Seq. proportion (% of total V3-V4)	Seq. proportion (% of total V4-V5)	Num. ASVs (V3-V4)	Num. ASVs (V4-V5)	ASVs ratio (V3-V4/V4-V5)
NB1-j_uncl	NB1-j_uncl	1	3	255	332	0.77
Alphaproteobacteria	Kiloniellaceae	1	2	37	73	0.51
Bacilli	Entomoplasmatales	1	2	4	3	1.33
Bacteroidia	Cyclobacteriaceae	1	2	125	116	1.08
Acidimicrobiia	Actinomarinales_uncl	1	1	48	40	1.20
Alphaproteobacteria	Clade_II	1	1	52	46	1.13
Alphaproteobacteria	Defluviicoccales_uncl	1	1	57	71	0.80
Alphaproteobacteria	Magnetospiraceae	1	1	69	81	0.85
Bacteroidia	Saprospiraceae	1	1	53	77	0.69
BD2-11_terrestrial_group	BD2-11_terrestrial_group_uncl	1	1	82	100	0.82
Gammaproteobacteria	Alteromonadaceae	1	1	28	8	3.50
Gammaproteobacteria	Halieaceae	1	1	87	51	1.71
Gammaproteobacteria	Spongiibacteraceae	1	1	62	50	1.24
Gammaproteobacteria	Thioglobaceae	1	1	15	7	2.14
Phycisphaerae	Phycisphaeraceae	1	1	117	206	0.57
Acidimicrobiia	Actinomarinaceae	1	0	3	3	1.00
Acidimicrobiia	Ilumatobacteraceae	1	0	20	16	1.25
Acidimicrobiia	Microtrichaceae	1	0	39	30	1.30

Class	Family	Seq. proportion (% of total V3-V4)	Seq. proportion (% of total V4-V5)	Num. ASVs (V3-V4)	Num. ASVs (V4-V5)	ASVs ratio (V3-V4/V4-V5)
Alphaproteobacteria	AEGEAN-169_marine_group	1	0	31	18	1.72
Alphaproteobacteria	SAR116_clade	1	0	13	8	1.63
Gammaproteobacteria	Arenicellaceae	1	0	25	11	2.27
Gammaproteobacteria	Cycloclasticaceae	1	0	3	1	3.00
Gammaproteobacteria	Methylophagaceae	1	0	8	6	1.33
Gammaproteobacteria	Methylophilaceae	1	0	35	18	1.94
Gammaproteobacteria	Pseudohongiellaceae	1	0	36	28	1.29
Gammaproteobacteria	Saccharospirillaceae	1	0	20	9	2.22
Gammaproteobacteria	UBA10353_marine_group_uncl	1	0	43	26	1.65
Planctomycetes	Rubinisphaeraceae	1	0	19	14	1.36
Nitrososphaeria	Nitrosopumilaceae	0	7	2	165	0.01
Gammaproteobacteria	AT-s2-59_uncl	0	1	24	32	0.75
Gammaproteobacteria	BD7-8_uncl	0	1	24	11	2.18
Gammaproteobacteria	Gammaproteobacteria_uncl	0	1	49	58	0.84
Gammaproteobacteria	Kangiellaceae	0	1	14	10	1.40
Gammaproteobacteria	Unknown_Family	0	1	98	80	1.23

Supplementary Table 4. Specificities of rRNA-targeting oligonucleotide probes used during CARD-FISH. FA: formamide concentration in the hybridization buffer.

Probe name	Target group	Sequence (5’-3’)	FA (%)	Reference
PSA184	*Alteromonadaceae, Colwelliaceae, Pseudoalteromonadaceae*	CCC CTT TGG TCC GTA GAC	30	(Eilers et al., 2000)
GAM42a	*Gammaproteobacteria*	GCC TTC CCA CAT CGT TT	35	(Manz et al., 1992)
BET421	competitor for GAM42a	GCC TTC CCA CTT CGT TT	35	(Manz et al., 1992)
POL740	*Polaribacter*	CCC TCA GCG TCA GTA CAT ACG T	35	(Malmstrom et al., 2007)
CF968	*Bacteroidia*	GGT AAG GTT CCT CGC GTA	55	(Acinas et al., 2015)
SAR11-152R	SAR11 clade	TTAGCACAAGTTTCCYCGTGT	25	(Morris et al. 2002)
SAR11-441R	SAR11 clade	TACAGTCATTTTCTTCCCCGAC	25	(Morris et al. 2002)
SAR11-441Rmod	SAR11 clade	TACCGTCATTTTCTTCCCCGAC	25	(Gomez-Pereira et al., 2013)
SAR11-542R	SAR11 clade	TCCGAACTACGCTAGGTC	25	(Morris et al. 2002)
SAR11-732R	SAR11 clade	GTCAGTAATGATCCAGAAAGYTG	25	(Morris et al. 2002)
SAR11-487Rmodif	SAR11 clade	CGGACCTTCTTATTCGGG	25	(Gomez-Pereira et al., 2013)
SAR11-487_h3	SAR11 clade	CGGCTGCTGGCACGAAGTTAGC	25	(Gomez-Pereira et al., 2013)
